# Building coherent monitoring and evaluation plans with the Evaluation Planning Tool for global health

**DOI:** 10.1080/16549716.2022.2067396

**Published:** 2022-09-13

**Authors:** Timothy Roberton, Talata Sawadogo-Lewis

**Affiliations:** Department of International Health, Johns Hopkins Bloomberg School of Public Health, Baltimore, MD, USA

**Keywords:** Evaluation, monitoring and evaluation, M&E, non-governmental organizations, NGOs, donors, planning

## Abstract

Practitioners in global health are called to monitor and evaluate their projects. This keeps projects on track, it meets donor and public demand, and it is a key mechanism by which global health organizations hold themselves accountable and improve their community of practice. However, monitoring and evaluation (M&E) is time- and resource-consuming, bringing into question whether the effort expended on M&E is worth it. While there has been a shift towards emphasizing the learning aspect of M&E, non-governmental organizations (NGOs) and other actors still struggle to get value from their efforts. One reason for this is that M&E plans are often not coherent or employed to their full potential. Theories of change, indicator lists, and data collection become a series of disjointed efforts that do not tie together. They become tick-the-box exercises to satisfy donors rather than a logical approach to draw meaningful findings for stakeholders, governments, and local communities. In this paper, we propose a step-by-step approach to utilizing M&E tools to their fullest potential, including: (1) a clearly defined theory of change that captures all program pathways and shows all intermediate objectives needed to achieve impact, (2) indicators which directly reflect the intermediate and ultimate objectives in the theory of change, and (3) a data collection plan which includes appropriate methods to measure indicators and address the questions stakeholders want answered. We make the case for a simpler, more coherent approach to M&E and propose a new tool to help practitioners more easily develop evaluation plans that are rigorous, practical, and worth the effort.

## Background

In recent years, non-governmental organizations (NGOs) and other global health actors have adopted a common set of tools for designing, monitoring, and evaluating projects [1]. These tools are used for articulating in advance how a project is expected to achieve its impact, for tracking the progress of project activities, and for determining whether and how a project improved population health [2,3]. NGOs use tools such as a theory of change, a logical framework matrix, an indicator list, and an activity timeline [4]. These tools help to set expectations for a project, so that the project’s implementors, funders, and other stakeholders have a shared understanding of what will and did happen. The composition of these tools for monitoring and evaluation (M&E) has evolved over the years, but their purpose has stayed the same. Most M&E plans include a hierarchy of objectives and activities, and a list of indicators to measure progress towards those objectives and activities.

There are good reasons for using M&E tools and committing to M&E in general. Global health actors have a responsibility to use resources effectively, which means proposing and implementing projects that actually achieve impact [5,6]. At the outset of a project, NGOs need a clear rationale for why a project will work. Throughout a project, NGOs need to monitor a project’s progress to ensure it is on track and change course if needed. And at the end of a project, NGOs need to understand and report on a project’s impact. Practitioners *should* be tracking and questioning the success of their projects, and M&E tools give organizations a standard approach to do this.

However, despite consensus on the need for and potential of M&E, there is a sense that the promise of M&E is still not yet being met; the *benefit* gained from M&E doesn’t match the *cost* of undertaking it [7,8]. The burden placed on NGOs to report against a monitoring plan can be overwhelming [9]. Data collection takes time, money, and technical capacity, and typically requires a dedicated team or organizational department [10]. The rise of digital technologies offers opportunities for more extensive data collection, but also raises expectations. The burden of M&E is often transferred to frontline workers or the community itself, with health workers or other staff required to expend undue energy on reporting, consuming their own time for the sake of the project.

Moreover, for all the effort put towards it, M&E rarely produces the full value that was expected from it [11]. The tools are used, but the findings can be limited. Often the motivation for adopting M&E tools is external, to satisfy a proposal submission or reporting requirements. The tools become tick-the-box exercises, set to the side except when reporting to a donor; part of the trend of the ‘commodification’ of projects [12]. Even when M&E tools are adopted willingly and with good intentions, the task of revisiting and updating the tools is superseded by the demands of the project itself. In the end, M&E becomes one more thing to implement.

One reason why NGOs do not get full value from M&E tools is that the information they generate does not ‘come together’. There is an over-emphasis on indicators, without an understanding of what the indicators are measuring or what the reported numbers ultimately say about the success or failure of the project. Theories of change (ToC) are used at the outset of a project – to show what the project is meant to achieve and how it will do it – but rarely during or beyond the project to reflect on its implementation or implicit assumptions. Indicators are disconnected from ToCs, and when the reported indicator numbers fall short of targets, they are seen as a collection of metrics, rather than what they should be: a representation of whether a project’s intended ‘impact pathways’ were successful.

Good M&E takes a lot to get right: resources, technical capacity, willing community collaboration, participatory approaches, contextual awareness; and stakeholders must actually want to generate findings – even if they are not flattering. But if the overarching plan for M&E is not logically sound, it will not matter how great the effort. In this paper, we lay out a process for creating an evaluation plan that has a coherent logic, and we describe a new, online tool that helps NGOs do this quickly and efficiently.

### What it takes to succeed: interdependent program pathways

It is rarely the case that one single project activity, by itself, will improve population health. Projects have many moving parts, and these parts need to work together for a project to achieve its impact. This is especially true in public health, where population outcomes are a result of complex factors, where service delivery depends on multiple health system components, and where the determinants of health span many sectors [13,14]. For example, imagine a project that aims to train health workers in order to increase the number of people being treated for malaria. Training health workers will increase the availability of services, and it may increase demand for services (if people hear about new services being offered), but if other factors prevent people from coming to facilities (long distances, prohibitively expensive fees), or limit the ability of health workers to provide effective care (no diagnostic tests or antimalarials at facilities), the project may not increase malaria treatment at all.

Many projects are like this, with multiple activities each necessary for overall success. A project may meet some of its intermediate objectives, but it will fail to achieve impact if another intermediate objective was not met [15]. We call these inter-related activities ‘program pathways’ [16]. The mistake is to think that each pathway is ‘sufficient’, whereas in reality each pathway is typically only ‘necessary’, and the overall contribution is only achieved if all activities are successfully implemented.

Sometimes the required activities or conditions for success are *external* to the project. For example, an NGO may train and equip health workers to diagnose malaria, but if there are no malaria treatment drugs at health facilities, the project is unlikely to improve coverage of malaria treatment or reduce malaria mortality (because people will be *diagnosed*, but not appropriately *treated*). If project planners want their project to succeed, they must consider all the factors needed for the project to have an impact: the factors that the project itself will address, *and* the factors that are external to the project but which the project depends on [15]. Designing projects to succeed involves identifying all the pathways needed for a project to achieve impact. Planners typically do this in a ‘theory of change’ or ‘impact model’.

### Articulating program pathways with a theory of change

Theories of change (ToC) are a common tool by which practitioners articulate how a project will achieve its impact [17]. ToCs consist of boxes and arrows, showing how one factor affects another. A clear, well-articulated ToC reflects a logical, well-considered project. People who teach writing sometimes say that ‘clear writing is clear thinking’. If writing is confusing, it can reflect an underlying confusion about what the author is trying to say. The same is true for project design and theories of change. A good ToC reflects a clear understanding of how a project will achieve its impact.

While most ToCs give a general idea about a project, some ToCs are insufficiently clear about the causal pathways from project activities to impact. While these ToCs are not wrong *per se*, they simply do not capture enough detail to show the true pathways necessary to achieve impact. Typically, boxes and arrows are either unclear, unspecified, or missing altogether. In other words, these ToCs only include a subset of the needed pathways and omit other factors such as those external to the project. The second issue is that ToCs are not employed to their full potential. In many cases, NGOs will create a ToC at the outset of the project – to articulate the vision or rationale for the project, or because a donor requires it as part of a funding application. But the most important reason for using a ToC is to ground the ongoing implementation and evaluation of a project, from beginning to end [18]. One of the ways that a ToC should do this is by serving as a framework for indicator selection, measurement, and analysis.

### Using a theory of change to guide measurement and determine indicators

A good ToC will articulate all the intermediate objectives that must be met for a project to achieve its intended impact. Thus, to understand if the project is succeeding, an NGO should measure all these intermediate objectives to see what changes and what does not. This can be done *throughout* the project for course correction and *after* the project as part of a summative evaluation to understand why the project achieved or did not achieve its objectives. Either way, a project’s attainment of objectives needs to be measured. Indicators and a measurement plan serve this purpose: to measure the activities, outputs, outcomes, and impact of a project.

Indicators should be chosen because they measure items in the ToC. Often, they are not. Some donors have pre-specified indicators of interest – chosen for their own internal purposes – and they require NGOs to measure these indicators. Some NGOs themselves have a pre-existing ‘library’ of indicators and choose indicators from this library. Instead, indicators should be chosen at a later stage, after the project’s impact pathways have been articulated. In this way, practitioners can build a logical, coherent plan in which the measurement of each indicator directly contributes to an understanding of whether, and how, a project was successful.

Without an exact link between indicators and a ToC, there is the risk that the indicators will not enable a full picture of the project’s impact pathways. If, at the end of a project, the indicators show that the project did not achieve impact, we want to know *why*. Unless we can interrogate a project’s full impact pathway – from activities, to intermediate objectives, to impact – it will be hard to know which link in the chain was the bottleneck. Was it because the project’s activities were not implemented successfully? If so, this would suggest a problem with project management or other implementation issues. Or was it because the activities did not lead, as anticipated, to one of the intermediate objectives? This would suggest a flaw in the project’s assumptions, perhaps a weakness in project design. Or was it because one of the other, external factors or dependencies of the project was not in place? This might indicate a contextual constraint and raise issues about the siloed nature of the project. Whatever the reason, it is important for everyone – the project stakeholders, the donor, and the global health community – to learn from the project in this way. And to do this, an M&E plan needs to include indicators that measure all the items in the ToC.

An exception to this is if the NGO or donor only wants to understand if the project was implemented as intended, and not whether the project achieved impact. Measuring indicators related to activities and processes will allow practitioners to answer the question, ‘Did we do what we said we would do?’ In many cases, this may be enough. Measuring more downstream indicators around health system functioning and population health, in a rigorous way, can take significant resources. Unless an NGO or donor is willing to invest appropriate resources to collect this population-level information, it may be sufficient to simply know whether the project was implemented as intended. In which case, an indicator matrix may only need to capture the upstream items in a ToC, those related to activities and their immediate outputs.

### Understanding trade-offs in data collection methods

Once indicators have been selected, the next step is to establish a data collection plan. Collecting data can be burdensome and expensive, especially for indicators that are best measured at facility or household level, which are typically those further ‘downstream’ in a ToC, such as those that measure intermediate objectives and impact. For example, measuring coverage of services typically requires a household survey with a representative sample of the population [10]. Measuring health status indicators, such as nutritional status or child mortality, can be even more complex, requiring heavy tools and resource-intensive data collection methods. For example, it is extremely difficult to measure neonatal or maternal mortality; the methods and sample sizes needed to achieve an accurate measurement are prohibitive for most NGOs. Practitioners must carefully weigh how valuable data is to their M&E plan when considering which indicators are worth the effort. As discussed above, it may be sufficient for stakeholders to simply understand if a project was implemented as intended, and this may only require measuring activity- and output-level indicators.

Some type of indicators can be measured, or at least calculated, in multiple ways. For example, there are different ways of measuring quality of care: using vignettes, record review, observation of consultations, or observation and re-examination. With each of these methods comes a trade-off between resources required (time, equipment, technical skills) and the accuracy and scope of the resulting measurement. Other indicators can be calculated using mathematical modeling. For example, the Lives Saved Tool is often used to calculate mortality indicators from a set of coverage indicators [19]. These options, while not the gold standard, may represent an appropriate balance between M&E *effort required* and *value gained*. Once decisions around data collection methods have been made, a full data collection plan will include the timing, frequency, location, and people responsible for each data collection activity.

### Building a cohesive, logical evaluation plan

An evaluation plan must bring all these pieces together – a theory of change, an indicator matrix, and a data collection timeline. Rather than each piece standing on its own, they should be logically connected. The indicators must reflect the program pathways in the theory of change, the data sources must match the indicators, and the timing and frequency of data collection must allow evaluators to answer the questions that stakeholders want answered.

In sum, we see three key steps to building a cohesive plan:

1. Develop a theory of change. Articulate all the pathways needed for a project to be successful, including all necessary intermediate objectives – the ‘links in the chain’ – for the project to achieve impact. Items in the ToC should be organized in a hierarchy (for example, outputs, outcomes, and impact, or immediate outcomes, intermediate outcomes, and ultimate outcomes.) Exact terminology can match donor requirements or be customized, but in any case, all the intermediate objectives needed for success must be identified.
2. Select indicators. Assign appropriate indicators to each of the items in the ToC and organize them according to the same levels or hierarchy as in the ToC. The extent of this indicator list should be informed by the type of inferences that stakeholders seek to draw from the evaluation; whether to measure only activity- and output-level indicators (to understand project implementation) or to also measure outcome- and impact-level indicators (to understand the impact of the project on population health).
3. Select data sources. Identify the data collection methods that will be used to measure each indicator, understanding the resources required, accuracy, and limitations of each method. From this, generate a short list of data sources that will allow for measuring, or modeling, all indicators. Determine the timing, frequency, location, and people responsible for each of the data collection methods.

Although we lay out these steps sequentially, it may take some iteration and back-and-forth to build a plan that fits together coherently. These steps also begin at the stage where the project itself has been thought through and the activities determined. It may be necessary to revisit the activities if the M&E planning process raises new information or reveals tenuous assumptions. For more rigorous, large-scale impact evaluations (‘plausibility’ and ‘probability’ evaluations) [20], other study design questions must be considered, including whether there will be a comparison area, how comparison areas will be selected or randomized, and appropriate sample sizes and sampling methodologies.

### The Evaluation Planning Tool

One of the barriers to undertaking the steps listed above is the logistics of mapping out a ToC, assigning indicators, and listing and consolidating data sources. It is fiddly work, and it can be an administrative challenge to keep everything organized with the usual suite of workplace tools (Word, Excel, PowerPoint). To fill this gap, we created a web application called the *Evaluation Planning Tool* (EPT – evaluationplanningtool.org). The tool consists of three panels: one for developing the theory of change, one for selecting indicators, and one for assigning data sources. The information populated in each of the panels connects to the other panels, with indicators being linked to the ToC, and data sources being linked to indicators. It is a quick, easy way for users to put together the core components of a robust evaluation plan.

Other software exists and is used by NGOs for M&E; for example, DevResults [21], Kinaki [22], and TolaData [23]. While these applications have tremendous functionality, including functionality for defining indicators, their scope is broader, being comprehensive management tools for organizing and visualizing data from any number of indicators. By contrast, the EPT is focused solely on the planning stage, and was specifically designed to enforce consistency between a theory of change, indicators, and data sources, with some novel features that we have not seen in other tools.

The centerpiece of the EPT is an interactive area for creating a visual impact model or ToC. Users can use this area to create and delete boxes, and drag-and-drop items to position them as necessary. The tool automatically updates the visual image, laying out the model in a logical format. This functionality alone makes it easier and quicker for users to create a theory of change in the EPT than in other tools. For example, in PowerPoint, or in an online design tool such as Figma, the addition of a new box requires manually adjusting arrows and other boxes – which is both tedious and finicky. The EPT’s algorithm automatically adjusts the arrows and boxes to lay out the visual image in the clearest way possible. Moreover, the tool’s structure helps models conform to a framework using headers (e.g. ‘inputs’, ‘activities’, etc.) which users can customize to suit their or their donor’s needs. Users can then add indicators to their plan and assign indicators to specific items in the ToC. Similarly, they can add data sources and determine the frequency at which data will be collected. Data sources can be assigned for each indicator.

Beyond the ease-of-use of the tool, the EPT has significant advantages. Being an online application, users can quickly access and use the tool in their web browser, without needing to download any software and without needing more than a cheap laptop. Although users do need an internet connection, the application is lightweight and does not require much bandwidth, so even users with a slow connection can still use the tool. The simple click-and-drag functionality means the learning curve is barely noticeable, with typical users able to navigate the tool within ten minutes. Users can collaborate on a shared plan and edits appear in real-time, similar to Google Docs. This promotes collaborative work and can ensure that stakeholders of all kinds can access and/or modify the plans as they are developed. The ease of modification removes the administrative barrier to modifying the ToC over the course of the project, which in turn encourages users to think of a ToC as a living document rather than a static exercise considered only at the start of the project.

The EPT has been available for NGOs and other stakeholders to use for three years now. Over 500 users have created an account, from organizations including NGOs, donors, universities, and government institutions. The feedback has been largely positive, confirming that the tool is indeed easy-to-use and that it meets a need as a way to quickly create an M&E plan with a strong internal logic. At Johns Hopkins University, we have used the tool in graduate courses on program evaluation and have seen first-hand the ease with which students are able to pick it up and use it to create theories of change and measurement plans.

We see potential for the tool be further embedded into NGO and donor workflows, although we expect such integrations to take time. An earlier iteration of the tool attempted to allow users to share evaluation plans directly with donors. However, given that donors have many different, extensive requirements, managing this information within the tool proved challenging and led to an overly complicated user interface. We opted instead to limit the tool to the core aspects of an M&E plan that we describe in this paper. We also chose not to include a ‘data’ component to the tool, which would potentially allow users to enter targets, baseline values, and updated values for indicators. This feature is typically included in other data management systems used by NGOs for collecting and maintaining M&E data, but it is not the focus of the EPT. However, future iterations of the EPT might allow users to overlay their data on an evaluation plan and embed their results into the tool directly.

## Conclusion

The global health community has well-established approaches for evaluating the impact of projects. These approaches make sense, but often the effort required to pursue them is great, while the value generated by them in practice is limited. Governments, organizations, and communities are doing the work, but not reaping the rewards. One opportunity to get greater utility from M&E is to ensure that all the pieces of an evaluation plan and monitoring strategy fit together; that the theory of change reflects the true program pathways needed to achieve impact; that the indicators are the right ones to measure outcomes along program pathways; and that the data collection methods enable timely, accurate, and feasible measurement of indicators. A cohesive evaluation will generate meaningful findings that *make sense* of what happened, not simply fill a reporting template. The Evaluation Planning Tool enforces a logical, cohesive linking of M&E components, and does so in an intuitive, easy-to-use interface.

## Paper context

The practice of global health frequently involves the monitoring and evaluating (M&E) of health projects. Significant effort is expended to develop M&E plans, collect data, and synthesize findings; yet as others have reported, the value gained from these efforts often does not match the resources invested. In this paper, we make the case for a simpler, more coherent approach to M&E and propose a new tool to help practitioners more easily develop robust evaluation plans.

## References

[1] Bach-Mortensen AM, Montgomery P. What are the barriers and facilitators for third sector organisations (non-profits) to evaluate their services? A systematic review. Syst Rev. 2018;7:23–28. 29357930 10.1186/s13643-018-0681-1 PMC5778760

[2] Christie CA. What guides evaluation? A study of how evaluation practice maps onto evaluation theory. New Dir. Eval. 2003;2003:7–36.

[3] Alkin MC, Vo AT, Hansen M. Using logic models to facilitate comparisons of evaluation theory. Eval. Program Plann. 2013;38:33. 22480564 10.1016/j.evalprogplan.2012.03.011

[4] Miller RL. Logic models: a useful way to study theories of evaluation practice? Eval. Program Plann. 2013;38:77–80. 22445548 10.1016/j.evalprogplan.2012.03.019

[5] Elbers W, Knippenberg L, Schulpen L. Trust or control? Private development cooperation at the crossroads. Public Adm. Dev. 2014;34:1–13.

[6] Koch DJ, Dreher A, Nunnenkamp P, et al. Keeping a low profile: what determines the allocation of aid by non-governmental organizations? World Dev. 2009;37:902–918.

[7] Tvedt T. Policy arena: the international aid system and the non-governmental organisations: a new research agenda. J. Int. Dev. 2006;18:677–690.

[8] Shukla A, Teedon P, Cornish F. Empty rituals? A qualitative study of users’ experience of monitoring & evaluation systems in HIV interventions in western India. Soc Sci Med. 2016;168:7–15. 27632362 10.1016/j.socscimed.2016.08.041

[9] Taylor J, Soal S. Measurement in developmental practice: from the mundane to the transformational. Fowler A, Malunga C. editors. NGO management. London: Routledge. 2010; pp. 324–336.

[10] Munos M. The RADAR coverage tool: developing a toolkit for rigorous household surveys for reproductive, maternal. Newborn, and Child Health & Nutr. Indic. 2021. 10.1080/16549716.2021.2006419 PMC9481084 36098955

[11] Wisniewski JM, Yeager VA, Diana ML, Hotchkiss DR. Exploring the barriers to rigorous monitoring and evaluation of health systems strengthening activities: qualitative evidence from international development partners. Int J Health Plann Manage. 2016;31(4):e302-e311. doi:10.1002/hpm.2339 26927839

[12] Freeman S, Schuller M. Aid projects: the effects of commodification and exchange. World Dev. 2020;126:104731.

[13] Paina L, Peters DH. Understanding pathways for scaling up health services through the lens of complex adaptive systems. Health Policy Plan. 2011;27:365–373. czr054-. 21821667 10.1093/heapol/czr054

[14] Adam T, de Savigny D. Systems thinking for strengthening health systems in LMICs: need for a paradigm shift. Health Policy Plan. 2012 Oct;27:iv1–3.PMID: 23014149 23014149 10.1093/heapol/czs084

[15] Victora CG, Schellenberg JA, Huicho L, et al. Context matters: interpreting impact findings in child survival evaluations. Health Policy Plan. 2005 Dec;20:i18–i31.PMID: 16306066 16306066 10.1093/heapol/czi050

[16] Bryce J, Victora CG, Habicht JP, et al. MCE-IMCI technical advisors. Programmatic pathways to child survival: results of a multi-country evaluation of integrated management of childhood illness. Health Policy Plan. 2005 Dec;20:i5–i17.PMID: 16306070 16306070 10.1093/heapol/czi055

[17] Breuer E, Lee L, De Silva M, et al. Using theory of change to design and evaluate public health interventions: a systematic review. Implement Sci. 2016 May 6;11:63. PMID: 27153985; PMCID: PMC4859947. 27153985 10.1186/s13012-016-0422-6 PMC4859947

[18] Luskin RJC, Ho T. Comparing the intended consequences of three theories of evaluation. Eval. Program Plann. 2013;38:61–66. 22459670 10.1016/j.evalprogplan.2012.03.015

[19] Walker N. The role of modeling in evaluation: using the lives saved tool to help answer core evaluation questions. 2021. 10.1080/16549716.2021.2006421 PMC9481079 36098950

[20] Habicht JP, Victora CG, Vaughan JP. Evaluation designs for adequacy, plausibility and probability of public health programme. Int J Epidemiol. 1999;28:10–18. 10195658 10.1093/ije/28.1.10

[21] DevResults. https://www.devresults.com [cited 2022 Feb 1]

[22] Kinaki. https://kinaki.ca [cited 2022 Feb 1]

[23] TolaData. https://www.toladata.com [cited 2022 Feb 1]

